# Downscaled and bias-corrected bioclimatic dataset for the Mediterranean Sea (2005–2099)

**DOI:** 10.1016/j.dib.2024.110846

**Published:** 2024-08-28

**Authors:** Marco Fianchini, Cosimo Solidoro, Donata Canu

**Affiliations:** a National Institute of Oceanography and Applied Geophysics - OGS, Via Beirut 2, 34151 Trieste - Trst, Italy; b Department of Life Sciences, University of Trieste, 34127, Trieste, Italy; c Department of Environmental Life Sciences, University of Udine, 33100, Udine, Italy

**Keywords:** Biogeochemical model, RCP8.5, Nutrients, Climate change, Bioclimatic variables

## Abstract

This dataset provides annual statistical descriptors (mean, minimum, maximum, range and standard deviation) of key biogeochemical and physical variables for the Mediterranean Sea. It covers the period 2005–2099 under a high emissions scenario (CMIP5-RCP8.5), with a spatial resolution of 1/24 degree (∼4 km²). The CMIP5-RCP8.5 scenario considers the current trajectory of increasing greenhouse gas emissions and population growth to the end of the century with nominal policies to reduce emissions. Variables include temperature, salinity, pH, water velocity, nutrients (NO_3_, PO_4_, NH_4_), dissolved inorganic carbon, oxygen, and net primary production. Data are available for both surface and at bathymetry level. The original projections were generated using OGSTM-BFM and MFS16 models at daily time and 1/16 degree grid resolution. These were downscaled to 1/24 degree resolution and bias corrected by quantile delta mapping using CMEMS reanalysis products from 2005 to 2020. The dataset is provided in a user-friendly format so that it is accessible for various ecological and environmental modelling applications.

Specifications Table

**Table:** 

Subject	Ecological Modelling.
Specific subject area	Mediterranean Sea Physics and Biogeochemistry annual statistical descriptors for the 21th century in the light of RCP8.5 scenario*.*
Type of data	Processed, netcdf format*.*
Data collection	All the processing was performed in Python 3.12.3 using the packages xarray, dask scipy, numpy, pandas, cmethods, copernicusmarine, ddsapi, on a High Performance Computing system (*Galileo100*, Cineca-Italy) using a total of 24 cores and 256GB RAM. Surface and bottom Reanalysis data at monthly resolution were obtained by directly downloading them through *copernicusmarine* API service (A detailed method to obtain bathymetry level data is provided at https://help.marine.copernicus.eu/en/articles/6049335-how-to-retrieve-a-variable-at-bathymetry-level-in-python). Future projected data for *RCP8.5* scenario at daily resolution were downloaded through *ddsapi,* averaged to monthly data and homogenised in terms of dimensions and variables names. Trilinear interpolation was performed to downscale projected data to Reanalysis spatial resolution and missing values were filled using *Nearest Neighbour* method, in a conservative approach. The same procedure used to extract bathymetry level variables as for Reanalysis data was used. Quantile Delta Mapping algorithm was applied to minimise distributional biases between projections and reanalysis time-series data using the 2005–2020 overlapping period as control*.*
Data source location	Raw data are stored and freely available (Creative Commons Attribution 4.0 International (CC BY 4.0) license) at: *CMCC DDS API:* https://dds.cmcc.it/#/dataset/medsea-cmip5-projections-biogeochemistry/ https://dds.cmcc.it/#/dataset/medsea-cmip5-projections-physics/ E.U. Copernicus Marine Service Information: https://doi.org/10.25423/CMCC/MEDSEA_MULTIYEAR_PHY_006_004_E3R1 https://doi.org/10.25423/cmcc/medsea_multiyear_bgc_006_008_medbfm3
Data accessibility	Data are publicly available at: Repository name: 2005–2099 High resolution bioclimatic variables for the surface and bottom of the Mediterranean Sea Data identification number: 10.5281/zenodo.12780160 Direct URL to data: https://zenodo.org/doi/10.5281/zenodo.12780160 Download requires no registration or any other procedure.
Related research article	*none.*

## 1 Value of the Data

• The data provide an extended set of annual projected key bioclimatic variables for the surface and bottom of the Mediterranean Sea for the 21th century at high spatial resolution.
• Bioclimatic variables at regional scale can be useful for a variety of ecological and environmental research applications, ranging from environmental management and risk assessment to biogeographic and species distribution modelling studies.
• The data follows the World Geodetic System standard (WGS84) and are ready-to-use.
• The OGSTM-BFM model provides accurate simulations of plankton productivity and biogeochemical cycles for the Mediterranean Sea.

## 2 Background

The Mediterranean Sea is characterised by its unique marine ecosystems and significant economic activities, including fishing and tourism. It has complex biogeochemical dynamics influenced by unique oceanographic and climatic conditions. Climate change is exacerbating these dynamics and affecting the availability and distribution of nutrients. The 1/16° resolution Mediterranean Forecasting System and the *OGSTM-BFM* model provide detailed insights into the biogeochemical present and future state of the basin under different emission scenarios [1,2]. However, the complexity and scale of the data hinder their widespread, practical application. This dataset addresses these challenges by providing annual statistical descriptors of key climate change indicators and nutrients. The dataset is obtained by harmonising, downscaling and bias-correcting the model simulations outputs [1]. The decision to focus on the *RCP8.5* scenario is because it can be considered a fairly realistic scenario compared to *RCP4.5* [3]. The result is a ready-to-use dataset containing variables for the surface and bathymetric levels [4]. With improved accessibility and interpretability, this derived dataset represents an optimised resource for biologists, ecological modellers and policy makers involved in marine conservation and climate resilience planning.

## 3 Data Description

The dataset is composed of 95 files in a single folder. Names are structured as ‘{*statistical_indicator*}_{*varname*}_{*layer*}.nc’ (e.g. mean_thetao_bottom.nc). Note that for Net Primary Production, there are layers reporting the vertical integration of 0–220 m depth layers named ‘{*statistical_indicator*}_{*nppv*}_integrated.nc’. See Table 1 for a complete list of files and variables.

**Table 1:** Description of the dataset files. Variable names, units, statistics and file names describe the content of the repository.

Long name	Variable name	Unit	Statistic	Filenames	
*Temperature*	*thetao*	*°C*	*min*	*max_thetao_surface.nc*	*max_thetao_bottom.nc*
			*max*	*range_thetao_surface.nc*	*range_thetao_bottom.nc*
			*range*	*std.dev_thetao_surface.nc*	*std.dev_thetao_bottom.nc*
			*std.dev*	*mean_thetao_surface.nc*	*mean_thetao_bottom.nc*
			*mean*	*min_thetao_surface.nc*	*min_thetao_bottom.nc*
*Salinity*	*so*	*PSU*	*min*	*min_so_surface.nc*	*min_so_bottom.nc*
			*max*	*max_so_surface.nc*	*max_so_bottom.nc*
			*range*	*range_so_surface.nc*	*range_so_bottom.nc*
			*std.dev*	*std.dev_so_surface.nc*	*std.dev_so_bottom.nc*
			*mean*	*mean_so_surface.nc*	*mean_so_bottom.nc*
*Water velocity*	*Wv*	*m/s*	*min*	*min_WV_surface.nc*	*min_WV_bottom.nc*
			*max*	*max_WV_surface.nc*	*max_WV_bottom.nc*
			*range*	*range_WV_surface.nc*	*range_WV_bottom.nc*
			*std.dev*	*std.dev_WV_surface.nc*	*std.dev_WV_bottom.nc*
			*mean*	*mean_WV_surface.nc*	*mean_WV_bottom.nc*
*pH*	*ph*	*.*	*min*	*min_ph_surface.nc*	*min_ph_bottom.nc*
			*max*	*max_ph_surface.nc*	*max_ph_bottom.nc*
			*range*	*range_ph_surface.nc*	*range_ph_bottom.nc*
			*std.dev*	*std.dev_ph_surface.nc*	*std.dev_ph_bottom.nc*
			*mean*	*mean_ph_surface.nc*	*mean_ph_bottom.nc*
*Ammonium*	*nh4*	*[mmol m-3]*	*min*	*min_NH4_surface.nc*	*min_NH4_bottom.nc*
			*max*	*max_NH4_surface.nc*	*max_NH4_bottom.nc*
			*range*	*range_NH4_surface.nc*	*range_NH4_bottom.nc*
			*std.dev*	*std.dev_NH4_surface.nc*	*std.dev_NH4_bottom.nc*
			*mean*	*mean_NH4_surface.nc*	*mean_NH4_bottom.nc*
*Nitrate*	*no3*	*[mmol m-3]*	*min*	*min_NO3_surface.nc*	*min_NO3_bottom.nc*
			*max*	*max_NO3_surface.nc*	*max_NO3_bottom.nc*
			*range*	*range_NO3_surface.nc*	*range_NO3_bottom.nc*
			*std.dev*	*std.dev_NO3_surface.nc*	*std.dev_NO3_bottom.nc*
			*mean*	*mean_NO3_surface.nc*	*mean_NO3_bottom.nc*
*Phosphate*	*po4*	*[mmol m-3]*	*min*	*min_PO4_surface.nc*	*min_PO4_bottom.nc*
			*max*	*max_PO4_surface.nc*	*max_PO4_bottom.nc*
			*range*	*range_PO4_surface.nc*	*range_PO4_bottom.nc*
			*std.dev*	*std.dev_PO4_surface.nc*	*std.dev_PO4_bottom.nc*
			*mean*	*mean_PO4_surface.nc*	*mean_PO4_bottom.nc*
*Dissolved Oxygen*	*o2*	*[mmol m-3]*	*min*	*min_o2_surface.nc*	*min_o2_bottom.nc*
			*max*	*max_o2_surface.nc*	*max_o2_bottom.nc*
			*range*	*range_o2_surface.nc*	*range_o2_bottom.nc*
			*std.dev*	*std.dev_o2_surface.nc*	*std.dev_o2_bottom.nc*
			*mean*	*mean_o2_surface.nc*	*mean_o2_bottom.nc*
*Net Primary Production*	*nppv*	*[mg m-2 day-1]*	*min*	*min_nppv_integrated.nc*	
			*max*	*max_nppv_integrated.nc*	
			*range*	*range_nppv_integrated.nc*	
			*std.dev*	*std.dev_nppv_integrated.nc*	
			*mean*	*mean_nppv_integrated.nc*	
*Dissolved Inorganic Carbon*	*dissic*	*[mol m-3]*	*min*	*min_DIC_surface.nc*	*min_DIC_bottom.nc*
			*max*	*max_DIC_surface.nc*	*max_DIC_bottom.nc*
			*range*	*range_DIC_surface.nc*	*range_DIC_bottom.nc*
			*std.dev*	*std.dev_DIC_surface.nc*	*std.dev_DIC_bottom.nc*
			*mean*	*mean_DIC_surface.nc*	*mean_DIC_bottom.nc*

**Fig. 1:**
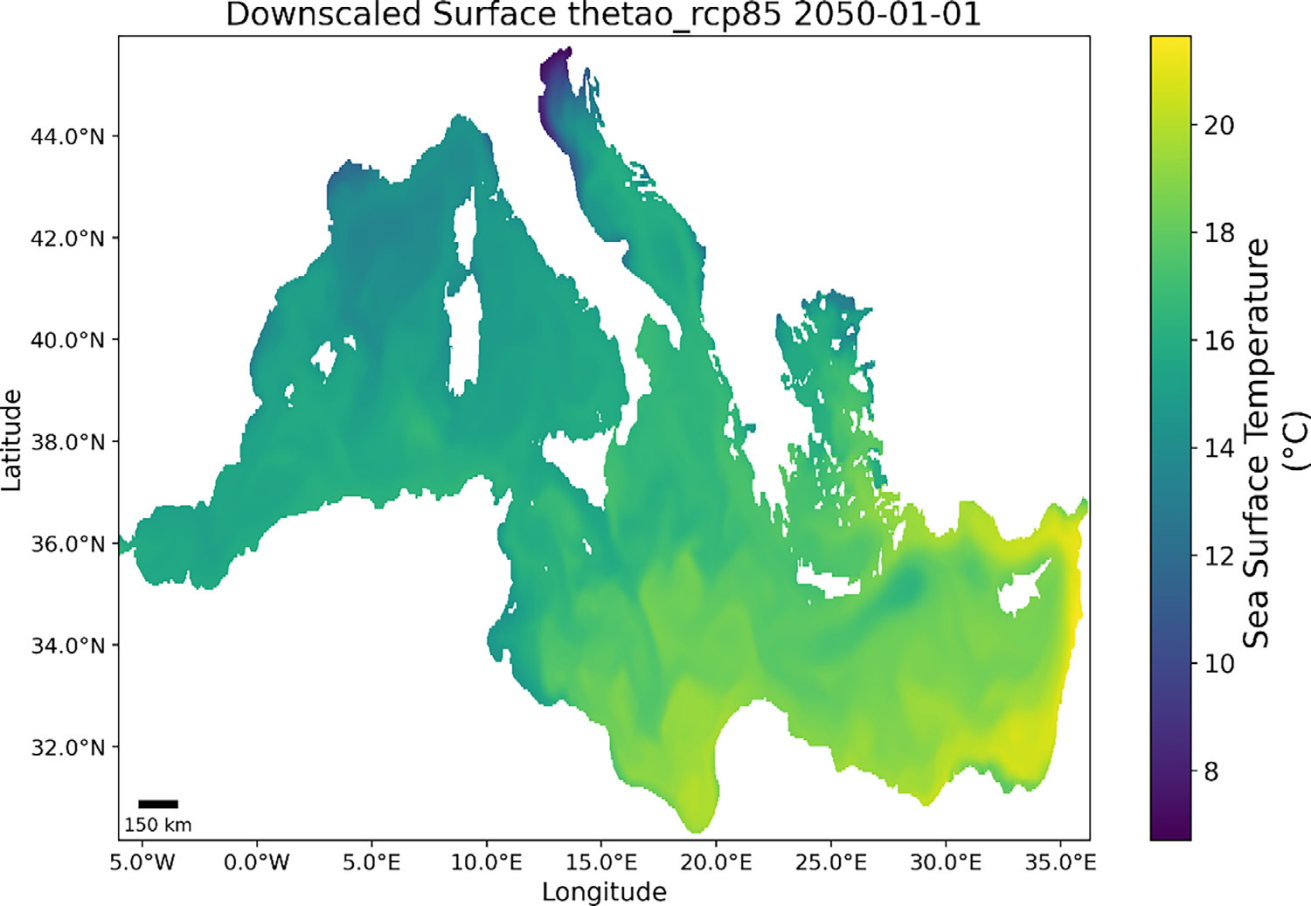
Projection of the mean surface temperature for the Mediterranean Sea in January 2050.

Each file covers the Mediterranean Sea (BBOX: *lon.min* = −6.00; *lon.max* = 36.28; *lat.min* = 30.21; *lat.max* = 45.97, CRS = *EPSG:4326*) for each year from 2005 to 2099. See Fig. 1 for an example*.*

## 4 Experimental Design, Materials and Methods

### 4.1 Computational environment

Processing was performed on *Galileo100* HPC system (Cineca-Italy) using 24 cores and 256GB RAM using Python language. Dependencies are: *copernicusmarine, ddsapi, xarray, dask, pandas, numpy, scipy, matplotlib, cmethods*.

### 4.2 Materials

The dataset was created using data from two primary sources: the European Union's Copernicus Marine Service Information (*CMEMS*) and the Centro Euro-Mediterraneo sui Cambiamenti Climatici (*CMCC*). The specific products used were:

1. Mediterranean Sea Biogeochemistry Reanalysis [5]. E.U. Copernicus Marine Service Information (*CMEMS*). Marine Data Store (*MDS*).
2. Mediterranean Sea Physical Reanalysis [6]. E.U. Copernicus Marine Service Information (*CMEMS*). Marine Data Store (*MDS*).
3. MEDSEA CMIP5 Projections Physics [7]. CMCC Data Delivery System (*DDS*).
4. MEDSEA CMIP5 Projections Biogeochemistry [1]. CMCC Data Delivery System (*DDS*).

### 4.3 The future of the Mediterranean in the light of the RCP 8.5 scenario

Simulations following the *RCP8.5* scenario predict significant changes in the Mediterranean by the end of the *21st* century:

1. Increased water temperature and stratification, reduction in vertical mixing and nutrient upwelling.
2. Reduced availability of phosphorus and nitrogen in the upper layers, limiting primary productivity and phytoplankton populations. The eastern Mediterranean could develop into oligotrophic conditions.
3. Reduced oxygen solubility and increased acidification due to higher CO_2_ uptake, affecting marine life, especially calcifying organisms.
4. Altered ecosystem composition and functionality with possible northward migration of species adapted to warm conditions and decline of less adaptable species.
5. Variable regional impacts, with potentially greater changes in the eastern Mediterranean compared to the western part.
6. Fluctuations in salinity due to changes in precipitation, river flows and evaporation rates, affecting water density and circulation patterns.

### 4.4 GIS modelling

All data processing was done using the native coordinate system World Geodetic System 1984 (WGS84) (*EPSG:4326*) to transform the regular 1/16° grid of the modelled data into the regular 1/24° grid of the reanalysis data. The distances are calculated “on the fly” using the *Haversine* formula. This approach minimises the risk of introducing integration and numerical errors associated with the planar projection and preserves the latitude/longitude trends as produced by the model simulations.

### 4.5 Experimental design and methods

Daily raw *RCP8.5* data from the *OGSTM-BFM* model for the period 2005–2100 with a grid resolution of 1/16 degree were processed to obtain bias-corrected statistical descriptors with a grid resolution of 1/24 degree and an annual time scale. Data processing included the following steps (package.function in brackets):

1. Monthly aggregation: the daily data were averaged monthly to reduce the data size and conform to the *CMEMS* reanalysis format.
2. Spatial downscaling: The data were downscaled to 1/24 degree using trilinear interpolation (*scipy.interpolate.interpn*) and masked (*numpy.where*) to exactly match the spatial grid of the *CMEMS* reanalysis.
3. Imputation of missing values: Missing values in the downscaled data set were filled using a nearest neighbour algorithm (*xarray.Dataset.ffill*), following a conservative approach. This ensured that the resulting dataset had the same number of values and the same spatial structure as the CMEMS reanalysis dataset while limiting extrapolation artifacts.
4. Bias correction: A Quantile Delta Mapping algorithm (*cmethods.adjust*, method = *quantile_delta_mapping*, quantiles = 10,000) was used to align projections to the trends in the *CMEMS* reanalysis time series [8]. The overlapping 2005–2020 period was used as a reference to fit a transfer function that maps the model's distribution to the reanalysis distribution while maintaining the relative changes projected by the model.
5. Calculation of statistical descriptors: Bias-corrected data were aggregated annually and five statistical indices were calculated: Minimum(*numpy.min*), Maximum(*numpy.max*), Mean(*numpy.mean*), Range(*numpy.range*) and Standard Deviation(*numpy.std*). The data were exported as *netcdf* files.

An example/template script for reproducing the entire process, starting with the download of the raw data, can be found in the Supplementary Material*.*

## Limitations

It is important to recognise that all model projections are subject to uncertainties. These can come from various sources, such as: Model structure, model parameterisation, uncertainties in initial conditions, uncertainties in boundary conditions and forcing functions (scenarios), numerical approximations. Usually the uncertainties in the initialisation dominate on a shorter time scale, the uncertainties in the scenarios are generally more important on a longer time scale, while the uncertainty in the choice of model parameterisation is always important. However, the results of a systematic global sensitivity analysis on the parameter uncertainties in a complex marine ecosystem model show that while the details of the time course of many variables are sensitive to the choice of parameter values, the integrated indicators are much more robust.

In this case, the importance of the atmospheric forcing and boundary conditions (i.e. the scenarios) should be emphasised, which can strongly influence the dynamics of the Mediterranean Sea, including vertical mixing, and thus the nutrient distribution and the dynamics of the lower trophic levels. Another point to consider is the spatial horizontal and vertical discretisation of the model. The *OGSTM-BFM* model focuses on resolving the dynamics of the low trophic levels in the euphotic zone at high resolution, while the representation of the processes in deep waters and near the bottom is less detailed. A final consideration concerns the downscaling and bias correction processes, which adjust and harmonise the dataset to the trends observed in the reanalysis, but may also introduce artefacts and smooth environmental signals. Considering all these factors, the dataset is well suited for studies at regional and sub-regional scales, but should be used with caution for applications at scales below grid resolution.

## Ethics Statement

The authors have read the ethical requirements for publication in Data in Brief and comply with them. They confirm that the present work does not include human subjects, animal experiments or data from social media platforms.

## CRediT authorship contribution statement

**Marco Fianchini:** Conceptualization, Methodology, Investigation, Data curation, Visualization, Writing – original draft, Writing – review & editing. **Cosimo Solidoro:** Conceptualization, Methodology, Validation, Writing – review & editing. **Donata Canu:** Conceptualization, Methodology, Validation, Writing – review & editing.

## Data Availability

2005–2099 High resolution bioclimatic variables for the surface and bottom of the Mediterranean Sea. (Original data) (Zenodo).
